# Transplantation of ACE2^-^ Mesenchymal Stem Cells Improves the Outcome of Patients with COVID-19 Pneumonia

**DOI:** 10.14336/AD.2020.0228

**Published:** 2020-03-09

**Authors:** Zikuan Leng, Rongjia Zhu, Wei Hou, Yingmei Feng, Yanlei Yang, Qin Han, Guangliang Shan, Fanyan Meng, Dongshu Du, Shihua Wang, Junfen Fan, Wenjing Wang, Luchan Deng, Hongbo Shi, Hongjun Li, Zhongjie Hu, Fengchun Zhang, Jinming Gao, Hongjian Liu, Xiaoxia Li, Yangyang Zhao, Kan Yin, Xijing He, Zhengchao Gao, Yibin Wang, Bo Yang, Ronghua Jin, Ilia Stambler, Lee Wei Lim, Huanxing Su, Alexey Moskalev, Antonio Cano, Sasanka Chakrabarti, Kyung-Jin Min, Georgina Ellison-Hughes, Calogero Caruso, Kunlin Jin, Robert Chunhua Zhao

**Affiliations:** ^1^School of Life Sciences, Shanghai University, Shanghai, China.; ^2^Institute of Basic Medical Sciences Chinese Academy of Medical Sciences, School of Basic Medicine Peking Union Medical College, Beijing, China.; ^3^Beijing YouAn Hospital, Capital Medical University, Beijing, China.; ^4^Department of Rheumatology and Clinical Immunology, Peking Union Medical College Hospital, Chinese Academy of Medical Sciences and Peking Union Medical College, Beijing, China.; ^5^Department of Orthopaedics, the First Affiliated Hospital of Zhengzhou University, Zhengzhou, China.; ^6^Institute of Stem Cell and Regeneration Medicine, School of Basic Medicine, Qingdao University, Shandong, China.; ^7^Department of Orthopaedics, the Second Affiliated Hospital of Xi’an Jiaotong University, Xi’an, China.; ^8^Department of Neurosurgery, the First Affiliated Hospital of Zhengzhou University, Zhengzhou, China.; ^9^The Executive Committee on Anti-aging and Disease Prevention in the framework of Science and Technology, Pharmacology and Medicine Themes under an Interactive Atlas along the Silk Roads, UNESCO, Paris, France.; ^10^International Society on Aging and Disease, Fort Worth, Texas, USA.; ^11^The Geriatric Medical Center "Shmuel Harofe", Beer Yaakov, affiliated to Sackler School of Medicine, Tel-Aviv University, Tel-Aviv, Israel.; ^12^School of Biomedical Sciences, Li Ka Shing Faculty of Medicine, University of Hong Kong, Hong Kong, China.; ^13^Institute of Chinese Medical Science, University of Macau, Taipa, Macau, China.; ^14^Institute of Biology, Komi Science Center of Russian Academy of Sciences, Syktyvkar, Russia.; ^15^Department of Pediatrics, Obstetrics and Gynecology, University of Valencia, Valencia, Spain.; ^16^Maharishi Markandeshwar Deemed University, Mullana-Ambala, India.; ^17^Department of Biological Sciences, Inha University, Incheon, South Korea.; ^18^Faculty of Life Sciences & Medicine, King's College London, London, UK.; ^19^Department of Biomedicine, Neuroscience and Advanced Diagnostics, University of Palermo, Palermo, Italy.; ^20^University of North Texas Health Science Center, Fort Worth, TX76107, USA.

**Keywords:** COVID-19, ACE2 negative, mesenchymal stem cells, cell transplantation, immunomodulation, function recovery

## Abstract

A coronavirus (HCoV-19) has caused the novel coronavirus disease (COVID-19) outbreak in Wuhan, China. Preventing and reversing the cytokine storm may be the key to save the patients with severe COVID-19 pneumonia. Mesenchymal stem cells (MSCs) have been shown to possess a comprehensive powerful immunomodulatory function. This study aims to investigate whether MSC transplantation improves the outcome of 7 enrolled patients with COVID-19 pneumonia in Beijing YouAn Hospital, China, from Jan 23, 2020 to Feb 16, 2020. The clinical outcomes, as well as changes of inflammatory and immune function levels and adverse effects of 7 enrolled patients were assessed for 14 days after MSC injection. MSCs could cure or significantly improve the functional outcomes of seven patients without observed adverse effects. The pulmonary function and symptoms of these seven patients were significantly improved in 2 days after MSC transplantation. Among them, two common and one severe patient were recovered and discharged in 10 days after treatment. After treatment, the peripheral lymphocytes were increased, the C-reactive protein decreased, and the overactivated cytokine-secreting immune cells CXCR3+CD4+ T cells, CXCR3+CD8+ T cells, and CXCR3+ NK cells disappeared in 3-6 days. In addition, a group of CD14+CD11c+CD11b^mid^ regulatory DC cell population dramatically increased. Meanwhile, the level of TNF-α was significantly decreased, while IL-10 increased in MSC treatment group compared to the placebo control group. Furthermore, the gene expression profile showed MSCs were ACE2^-^ and TMPRSS2^-^ which indicated MSCs are free from COVID-19 infection. Thus, the intravenous transplantation of MSCs was safe and effective for treatment in patients with COVID-19 pneumonia, especially for the patients in critically severe condition.

The novel coronavirus disease 2019 (COVID-19) has grown to be a global public health emergency since patients were first detected in Wuhan, China, in December 2019. Since then, the number of COVID-19 confirmed patients have sharply increased not only in China, but also worldwide, including Germany, South Korea, Vietnam, Singapore, Italy, and USA [1]. Currently, no specific drugs or vaccines are available to cure the patients with COVID-19 infection. Hence, there is a large unmet need for a safe and effective treatment for COVID-19 infected patients, especially the severe cases.

Several reports demonstrated that the first step of the HCoV-19 pathogenesis is that the virus specifically recognizes the angiotensin I converting enzyme 2 receptor (ACE2) by its spike protein [2-4]. ACE2-positive cells are infected by the HCoV-19, like SARS-2003[5,6]. In addition, a research team from Germany revealed that the cellular serine protease TMPRSS2 for HCoV-19 Spike protein priming is also essential for the host cell entry and spread [7], like the other coronavirus (i.e. SARS-2003) [8,9]. Unfortunately, the ACE2 receptor is widely distributed on the human cells surface, especially the alveolar type II cells (AT2) and capillary endothelium [10], and the AT2 cells highly express TMPRSS2 [9]. However, in the bone marrow, lymph nodes, thymus, and the spleen, immune cells, such as T and B lymphocytes, and macrophages are consistently negative for ACE2[10]. The findings suggest that immunological therapy may be used to treat the infected patients. However, the immunomodulatory capacity may be not strong enough, if only one or two immune factors were used, as the virus can stimulate a terrible cytokine storm in the lung, such as IL-2, IL-6, IL-7, GSCF, IP10, MCP1, MIP1A, and TNFα, followed by the edema, dysfunction of the air exchange, acute respiratory distress syndrome, acute cardiac injury and the secondary infection [11], which may lead to death. Therefore, avoiding the cytokine storm may be the key for the treatment of HCoV-19 infected patients. MSCs, owing to their powerful immunomodulatory ability, may have beneficial effects for preventing or attenuating the cytokine storm.

MSCs have been widely used in cell-based therapy, from basic research to clinical trials [12,13]. Safety and effectiveness have been clearly documented in many clinical trials, especially in the immune-mediated inflammatory diseases, such as graft versus-host disease (GVHD) [14] and systemic lypus erythematosus (SLE) [15]. MSCs play a positive role mainly in two ways, namely immunomodulatory effects and differentiation abilities [16]. MSCs can secrete many types of cytokines by paracrine secretion or make direct interactions with immune cells, leading to immunomodulation [17]. The immunomodulatory effects of MSCs are triggered further by the activation of TLR receptor in MSCs, which is stimulated by pathogen-associated molecules such as LPS or double-stranded RNA from virus [18,19], like the HCoV-19.

Here we conducted an MSC transplantation pilot study to explore their therapeutic potential for HCoV-19 infected patients. In addition, we also explored the underlying mechanisms using a 10× Genomics high throughput RNA sequencing clustering analysis on MSCs and mass cytometry.

## MATERIALS AND METHODS

### Study design

A pilot trial of intravenous MSC transplantation was performed on seven patients with COVID-19 infected pneumonia. The study was conducted in Beijing YouAn Hospital, Capital Medical University, China, and approved by the ethics committee of the hospital (LL-2020-013-K). The safety and scientific validity of this study “Clinical trials of mesenchymal stem cells for the treatment of pneumonitis caused by novel coronavirus” from Shanghai University/PUMC have been reviewed by the scientific committee at International Society on Aging and Disease (ISOAD) and issued in Chinese Clinical Trial Registry (ChiCTR2000029990).

**Table 1:** Clinical classification of the COVID-19 released by the National Health Commission of China.

Mild	Common	Severe	Critically severe
Mild clinical manifestation, None Imaging Performance	Fever, respiratory symptoms, pneumonia performance on X-ray or CT	Meet any of the followings: 1. Respiratory distress, RR ≥ 30/min; 2. Oxygen saturation ≤ 93% at rest state; 3. Arterial partial pressure of oxygen (PaO_2_) / Fraction of inspiration O_2_ (FiO_2_) ≤ 300mnHg, 1mmHg=0.133kPa	Meet any of the followings: 1. Respiratory failure needs mechanical ventilation; 2. Shock; 3. Combined with other organ failure, patients need ICU monitoring and treatment

### The patients

The patients were enrolled from Jan 23, 2020 to Jan 31, 2020. All enrolled patients were confirmed by the real-time reverse transcription polymerase chain reaction (RT-PCR) assay of HCoV-19 RNA in Chinese Center for Disease Control and Prevention using the protocol as described previously [11,20]. The sequences were as follows: forward primer 5′-TCAGAATGCCAATCTC CCCAAC-3′; reverse primer 5′-AAAGGTCCACCCGA TACATTGA-3′; and the probe 5′CY5CTAGTTACACT AGCCATCCTTACTGC-3′BHQ1.

We initially enrolled patients with COVID-19 (age 18-95 years) according to the guidance of National Health Commission of China (Table 1). If no improvement signs were observed under the standard treatments, the patient would be suggested to join this polit study. Patients were ineligible if they had been diagnosed with any kind of cancers or the doctor declared the situation to belong to the critically severe condition. We excluded patients who were participating in other clinical trials or who have participated in other clinical trials within 3 months.

### Cell preparation and transplantation

The clinical grade MSCs were supplied, for free, by Shanghai University, Qingdao Co-orient Watson Biotechnology group co. LTD and the Institute of Basic Medical Sciences, Chinese Academy of Medical Sciences. The cell product has been certified by the National Institutes for Food and Drug Control of China (authorization number: 2004L04792,2006L01037,CXSB1900004). Before the intravenous drip, MSCs were suspended in 100 ml of normal saline, and the total number of transplanted cells was calculated by 1 × 10^6^ cells per kilogram of weight. The window period for cell transplantation was defined as the time when symptoms or/and signs still were getting worse even as the expectant treatments were being conducted. The injection was performed about forty minutes with a speed of ~40 drops per minute.

The patients were assessed by the investigators through the 14-day observation after receiving the investigational product. The clinical, laboratory, and radiological outcomes were recorded and certified by a trained group of doctors. The detailed record included primary safety data (infusional and allergic reactions, secondary infection and life-threatening adverse events) and the primary efficacy data (the level of the cytokines variation, the level of C-reactive protein in plasma and the oxygen saturation). The secondary efficacy outcomes mainly included the total lymphocyte count and subpopulations, the chest CT, the respiratory rate, and the patient symptoms (especially the fever and shortness of breath). In addition, the therapeutic measures (i.e. antiviral medicine and respiratory support) and outcomes were also examined.

**Figure 1.**
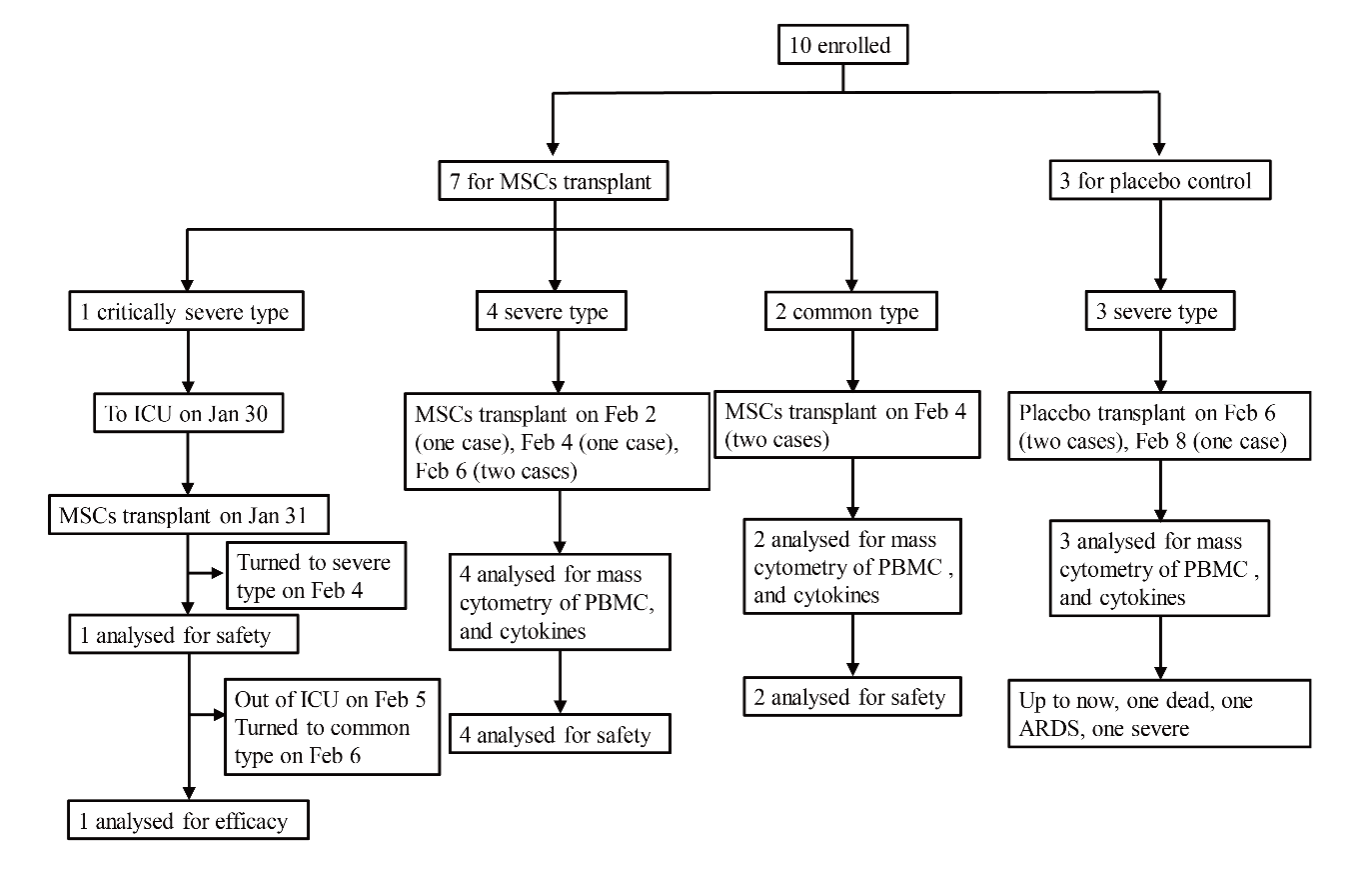
The flow chart of the cell transplantation treatment.

### Statistical analysis

MIMICS 21.0 (Interactive medical image control system of Materialise, Belgium) was used to evaluate the chest CT data. The analysis of Mass cytometry of the peripheral blood mononuclear cells is described in Supplementary 1. The analysis of the 10 x RNA-seq survey is described in Supplementary 2. Data were analyzed by SPSS software (SPSS 22.0). Differences between two groups were assessed using unpaired two-tailed t tests. Data involving more than two groups were assessed by analysis of variance (ANOVA). P values <0.05 indicated statistical significance.

**Table 2:** The general information of the enrolled patients.

	Case 1	Case 2	Case 3	Case 4	Case 5	Case 6	Case 7	Ctrl 1	Ctrl 2	Ctrl 3
Gender	M	F	F	F	M	M	M	F	F	F
Age (years)	65	63	65	51	57	45	53	75	74	46
COVID-19 type	Critically severe	Severe	Severe	Common	Common	Severe	Severe	Severe	Severe	Severe
Fever (?, baseline)	38.6	37.7	38.2	38.5	38.4	39.0	39.0	36.0	38.9	37.7
Shortness of breath	+++	+++	++	+	+	+++	+++	+++	++	+
Oxygen saturation at rest state	89%	93%	92%	95%	94%	92%	90%	91%	92%	93%
Cough, weak, poor appetite	++	+	++	+	++	++	++	+	++	+
Diarrhea	-	-	+	-	-	-	-	-	-	-
Date of diagnosed	Jan 23	Jan 27	Jan 25	Feb 3	Feb 2	Jan 27	Feb 3	Feb 3	Feb 6	Feb 5
Date of intervention (MSCs or Placebo)	Jan 31	Feb 2	Feb 4	Feb 4	Feb 4	Feb 6	Feb 6	Feb 8	Feb 6	Feb 6
Date of recovery	Feb 3	Feb 4	Feb 6 Discharged	Feb 6 Discharged	Feb 5 Discharged	Feb 7	Feb 7	Dead	ARDS	Stable

## RESULTS

### MSC treatment procedure and general patient information

This study was conducted from Jan 23, 2020, to Feb 16, 2020. Seven confirmed COVID-19 patients, including 1 critically severe type (patient 1), 4 severe types (patients 2, 3, 6, 7) and 2 common types (patients 4, 6) were enrolled for MSCs transplant, and three severe types were enrolled for placebo control. The timepoint of MSC transplantation for each patient is as shown in Figure 1. The general information of the 7 patients is listed in Table 2. Hitherto, the critically severe patient had completed the MSC treatment. This patient had a 10-year medical history of hypertension with the highest-level of 180/90 mmHg recorded. All the treatment information of the patients was collected.

#### The primary safety outcome

Before the MSC transplantation, the patients had symptoms of high fever (38.5? ± 0.5?), weakness, shortness of breath, and low oxygen saturation. However, 2~4 days after transplantation, all the symptoms disappeared in all the patients, the oxygen saturations rose to ≥ 95% at rest, without or with oxygen uptake (5 liters per minute). In addition, no acute infusion-related or allergic reactions were observed within two hours after transplantation. Similarly, no delayed hypersensitivity or secondary infections were detected after treatment.

The detailed diagnosis and treatment procedures of the critically severe patient are shown in Supplementary 3. The main symptoms and signs are shown in Table 3.

#### The efficacy outcome

The immunomodulating function of MSCs contributed to the main efficacy outcome and the transplantation of MSCs showed impressive positive results (Table 3). For the primary outcome in the critically severe patient 1, the plasma C-reaction protein level decreased from 105.5 g/L (Jan 30) to 10.1 g/L (Feb 13), which reached the highest level of 191.0 g/L on Feb 1, indicating that the inflammation status was alleviating quickly. The oxygen saturation, without supplementary oxygen, rose from 89% (Jan 31) to 98% (Feb 13), which indicated the pulmonary alveoli regained the air-change function.

The secondary outcomes were also improved (Table 4). Considering, for example, the critically severe patient 1, the lymphopenia was significantly improved after the cell transplantation. The patient was isolated in the hospital isolation ward with a history of hypertension and blood pressure reaching grade 3 hypertension. On Feb 1, biochemical indicators in the blood test showed that aspartic aminotransferase, creatine kinase activity and myoglobin increased sharply to 57 U/L, 513 U/L, and 138 ng/ml, respectively, indicating severe damage to the liver and myocardium. However, the levels of these functional biochemical indicators were decreased to normal reference values in 2~4 days after treatment (Table 4).

**Table 3:** Symptoms, signs and maximum body temperatures of the critically severe patient from Jan 21 to Feb 13, 2020. ICU: Intensive Care Unit; NA: Not Available.

	Home	Hospital	Hospital	ICU	ICU	ICU	ICU	ICU	Out of ICU	Hospital	Hospital
Date	Jan 21~22	Jan 23	Jan 24~29	Jan 30	Jan 31	Feb 1	Feb 2~3	Feb 4	Feb 5~8	Feb 9~12	Feb 13
Fever (?)	37.5	37.8	37.0~38.5	38.6	38.8	36.8	36.6~36.9	36.8	36.6~36.8	36.5~36.9	36.6
Shortness of breath	-	+	+	++	++++	++	+	-	-	-	-
Cough	+	+	+	++	++	+	+	-	-	-	-
Sputum	+	+	+	++	++	+	+	-	-	-	-
O_2_ saturation (without/with O_2_ uptake)	NA/NA	NA/NA	97% /NA	91%/ 95%	89% /94%	NA /98%	NA /97%	NA /96%	NA /97%	96% //NA	97% /NA
Respiratory rate	NA	23	23	27	33	22	22	21	20~22	20~22	21
Treatment (Basics-1: Antipyretic, antiviral and supportive therapy. Basics-2: antiviral and supportive therapy)	NA	NA	Basics-1	Basics-1; Mask O_2_ 5L/min	Basics-1; Mask O_2_ 10L/min; Cell transplant	Basics-1; Mask O_2_ 5L/min	Basics-2; Mask O_2_ 5L/min	Basics-2; Mask O_2_ 5L/min	Basics-2; Mask O_2_ 5L/min	Basics-2	Basics-2
RT-PCR of the virus	NA	Positive	NA	NA	NA	NA	NA	NA	Positive (Feb 6)	NA	Negative

**Figure 2.**
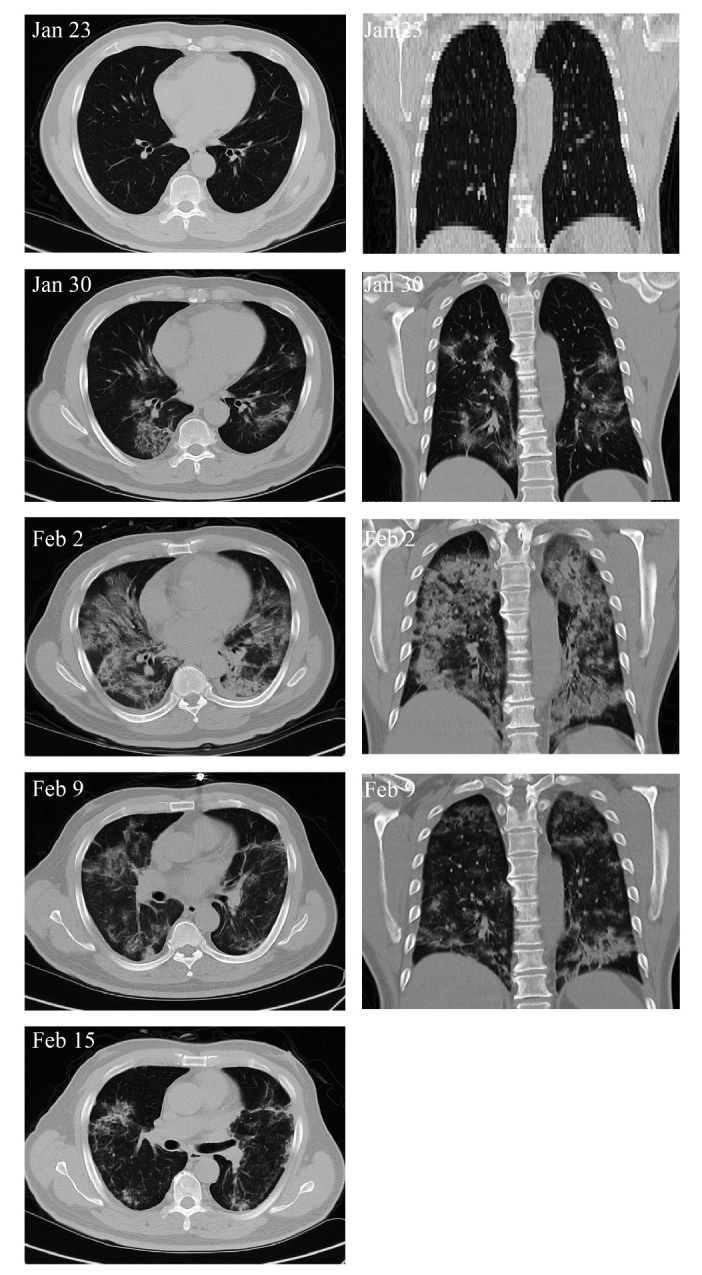
Chest computerized tomography (CT) images of the critically severe COVID-19 patient. On Jan 23, no pneumonia performance was observed. On Jan 30, ground-glass opacity and pneumonia infiltration occurred in multi-lobes of the double sides. Cell transplantation was performed on Jan 31. On Feb 2, the pneumonia invaded all through the whole lung. On Feb 9, the pneumonia infiltration faded away largely. On Feb 15, only little ground-glass opacity was residual locally.

**Figure 3.**
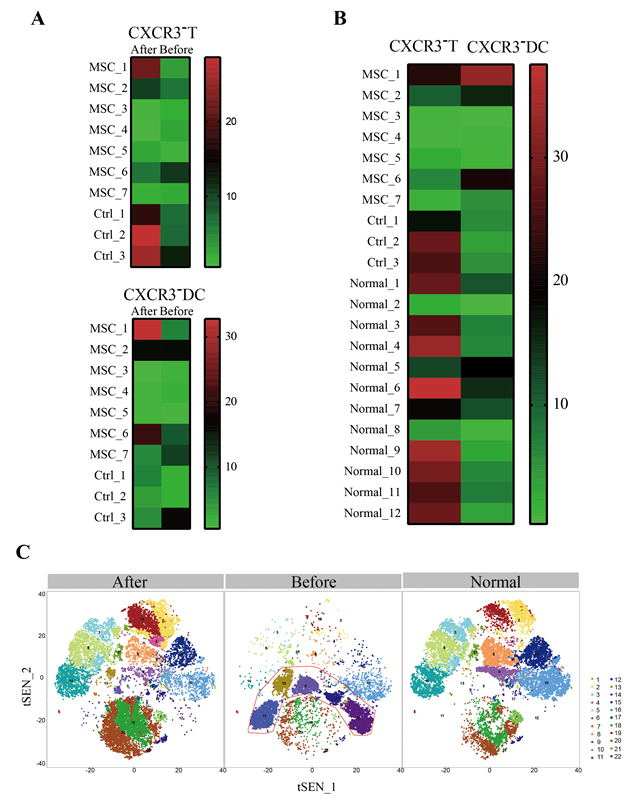
The profile of the peripheral blood mononuclear cells of patients. The mass cytometry results of peripheral blood mononuclear cells of the enrolled patients (A, B) and the critically severe patient (C). No increase of regulatory T cells (CXCR3-) or dendritic cells (DC, CXCR3-) for the two patients of common type (Patients 4 and 5, Figrue 3A). But in the severe patients, both the regulatory T cells and DC increased after the cell therapy, especially for the critically severe patient 1 (Figure 3B). Moreover, for the critically severe patient 1, before the MSC transplantation the percentages of overactivated CXCR3+CD4+ T cells (#9), CXCR3+CD8+ T cells (#17), and CXCR3+ NK cells (#12) in the patient’s PBMC were remarkably increased compared to the healthy control (Figure 3C). However, 6 days after MSC transplantation, the overactivated T cells and NK cells nearly disappeared and the numbers of the other cell subsets were almost reversed to the normal levels, especially the CD14+CD11c+CD11b^mid^ DC (#20) population. Normal: healthy individuals, MSCs: mesenchymal stem cells transplant group, Ctrl: placebo control group.

**Table 4:** The laboratory results of the critically severe patient. Red: the value was above the normal. Blue: the value was below the normal. NA: Not Available.

	Reference range	Jan 24	Jan 30	Jan 31	Feb 1	Feb 2	Feb 4	Feb 6	Feb 10	Feb 13
C-reactive protein (ng/mL)	< 3.00	2.20	105.50	NA	191.00	83.40	13.60	22.70	18.30	10.10
Absolute lymphocyte count (× 10^9^/L)	1.10-3.20	0.94	0.60	0.35	0.23	0.35	0.58	0.87	0.73	0.93
White-cell count (× 10^9^/L)	3.50-9.50	4.91	6.35	7.90	7.08	12.16	12.57	11.26	10.65	8.90
Absolute neutrophil count (× 10^9^/L)	1.80-6.30	3.43	5.43	7.28	6.63	11.33	11.10	9.43	9.18	7.08
Absolute monocyte count (× 10^9^/L)	0.10-0.60	0.38	0.25	0.17	0.13	0.35	0.61	0.52	0.48	0.56
Red-cell count (× 10^12^ /L)	4.30-5.80	4.69	4.68	4.66	4.78	4.73	4.75	5.16	4.69	4.53
Hemoglobin (g/L)	130.00-175.00	145.00	147.00	145.00	146.00	142.00	145.00	155.00	145.00	137.00
Platelet count (× 10^9^/L)	125.00-350.00	153.00	148.00	169.00	230.00	271.00	268.00	279.00	332.00	279.00
Absolute eosinophil count (× 10^9^/L)	0.02-0.52	0.02	0.02	0.02	0.02	0.02	0.05	0.15	0.14	0.14
Absolute basophilic count (× 10^9^/L)	0.00-0.06	0.02	0.01	0.02	0.02	0.02	0.06	0.10	0.03	0.04
Total bilirubin (μmol/L)	5.00-21.00	7.00	23.00	21.70	19.80	14.20	15.80	16.50	12.50	8.70
Albumin (g/L)	40.00-55.00	41.70	32.30	29.70	29.90	31.60	33.00	32.20	30.10	29.10
Aspartate amino transferase (U/L)	15.00-40.00	14.00	33.00	48.00	57.00	39.00	34.00	23.00	25.00	19.00
Fibrinogen (g/L)	2.00-4.00	2.44	4.24	NA	NA	4.73	NA	3.12	3.84	3.73
Procalcitonin (ng/mL)	< 0.10	0.11	0.12	NA	NA	NA	0.10	0.18	0.15	< 0.10
Creatine kinase isoenzymes (ng/mL)	< 3.60	0.90	0.12	NA	5.67	4.24	NA	0.88	0.90	0.61
Creatine kinase (U/L)	50.00-310.00	168.00	231.00	NA	513.00	316.00	NA	47.00	83.00	40.00
Glomerular filtration rate (ml/min)	> 90.00	81.30	68.00	89.60	99.00	104.00	92.50	108.10	97.10	94.10
Potassium (mmol/L)	3.50-5.30	3.61	2.74	3.00	3.42	3.47	4.18	4.36	4.69	4.61
Sodium (mmol/L)	137.00-147.00	138.50	132.60	129.50	132.80	136.90	135.80	133.80	134.10	137.70
Myoglobin (ng/mL)	16.00-96.00	53.00	80.00	NA	138.00	77.00	NA	62.00	60.00	43.00
Troponin (ng/mL)	< 0.056	0.10	0.07	NA	0.05	0.05	NA	0.02	0.04	0.04

On February 13, all the indexes reached normal levels, namely 19 U/L, 40 U/L, and 43 ng/ml, respectively. The respiratory rate was decreased to the normal range on the 4th day after MSC transplantation. Both fever and shortness of breath disappeared on the 4th day after MSCs transplantation. Chest CT imaging showed that the ground-glass opacity and pneumonia infiltration were largely reduced on the 9th day after MSC transplantation (Fig. 2).

#### HCoV-19 nucleic acid detection

RT-PCR analysis of HCoV-19 nucleic acid was performed before and after MSC transplantation. For the critically severe patient, before transplantation (Jan 23) and 6 days after transplantation (Feb 6), HCoV-19 nucleic acid was positive. 13 days after transplantation (Feb 13), HCoV-19 nucleic acid turned to be negative. The patients 3, 4,5 also turned to be negative for HCoV-19 nucleic acid until this report date.

#### Mass cytometry (CyTOF) analysis of the patients’ peripheral blood

To investigate the profile of the immune system constitution during MSC transplantation, we performed the CyTOF to analyze immune cells in the patients’ peripheral blood before and after transplantation. CyTOF revealed that there was nearly no increase of regulatory T cells (CXCR3^-^) or dendritic cells (DC, CXCR3^-^) for the two patients of common type (Patients 4 and 5). But in the severe patients, both the regulatory T cells and DC increased after the cell therapy, especially for the critically severe patient. Notably, no significant CXCR3^-^ DC enhancement was observed after placebo treatment in three severe control patients. Moreover, for the critically severe patient, before the MSC transplantation the percentages of CXCR3+CD4+ T cells, CXCR3+CD8+ T cells, and CXCR3+ NK cells in the patient’s PBMC were remarkably increased compared to the healthy control, which caused the inflammatory cytokine storm. However, 6 days after MSC transplantation, the overactivated T cells and NK cells nearly disappeared and the numbers of the other cell subpopulations were almost restored to the normal levels, especially the CD14+CD11c+CD11b^mid^ regulatory dendritic cell population (Fig. 3).

#### Serum Cytokine/Chemokine/Growth Factor Analysis

**Figure 4.**
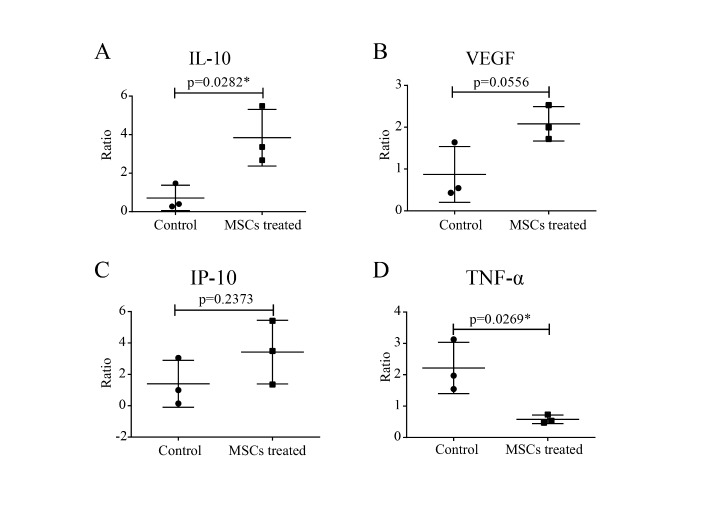
The profile of serum cytokine/chemokine/growth factors. The ratio of serum cytokines IL-10 (A), growth factor VEGF (B), the chemokine IP-10 (C) and TNF-α (D) before and after MSCs treatment were detected in severe patients compared with the control group without MSCs by panel assay analysis, respectively. Ctrl: placebo control group. P-values were determined using Student’s t-test. *P < 0.05.

After intravenous injection of MSCs, the decrease ratio of serum pro-inflammatory cytokine TNF-α before and after MSC treatment was significant (p<0.05). Meanwhile, the increase ratio of anti-inflammatory IL-10 (p<0.05) also showed remarkably in the MSC treatment group. The serum levels of chemokines like IP-10 and growth factor VEGF were both increased, though not significantly (Fig. 4).

#### 10 x RNA-seq analysis of transplanted MSCs

**Figure 5.**
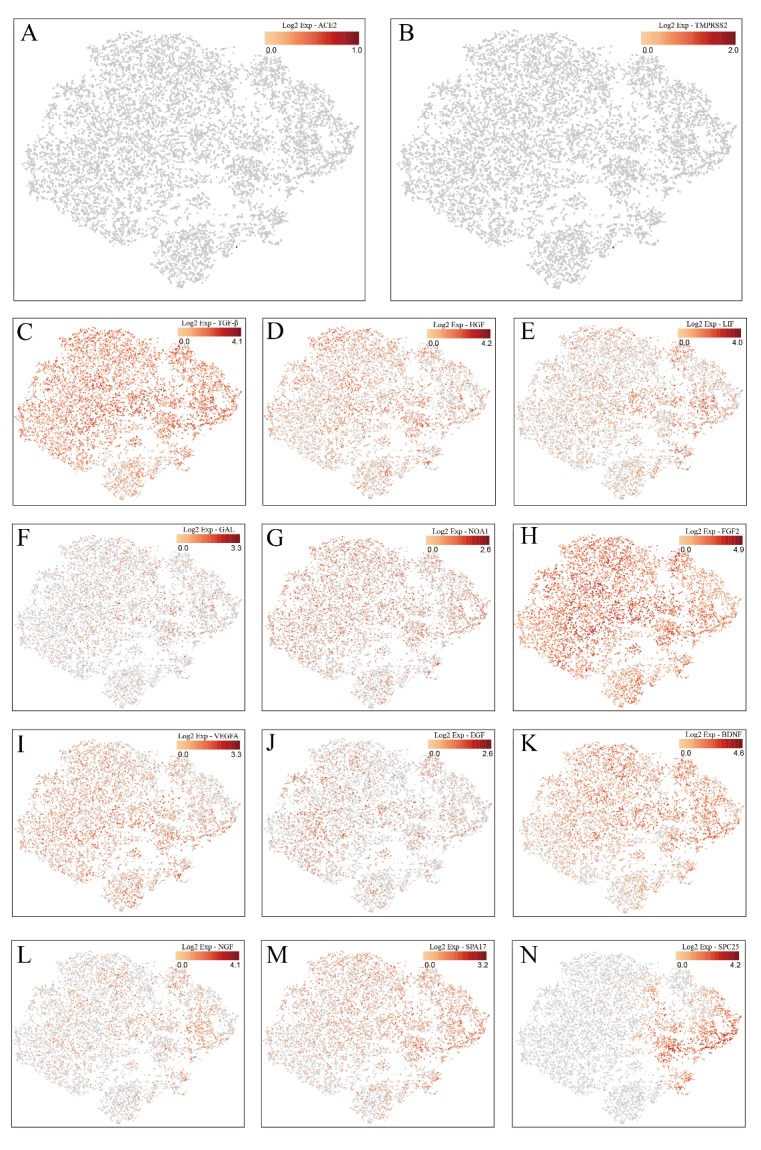
RNA-seq analysis of transplanted MSCs. The 10 x RNA-seq survey of MSCs genes expression: Both ACE2 (A) and TMPRSS2 (B) were rarely expressed. TGF-β (C), HGF (D), LIF (E), GAL (F), NOA1 (G), FGF (H), VEGF (I), EGF (J), BDNF (K), and NGF (L) were highly expressed, indicating the immunomodulatory function of MSCs. SPA (M) and SPC (N) were highly expressed, indicating MSCs possessed the ability to differentiate into the alveolar epithelial cells II. One point represented one cell, and red and gray color showed high expression and low expression, respectively.

To further elucidate the mechanisms underlying MSC-mediated protection for COVID-19 infected patients, we performed the 10 x RNA-seq survey for transplanted MSCs. The 10 x RNA-seq survey captured 12,500 MSCs which were then sequenced with 881,215,280 raw reads totally (Supplementary Fig. 2). The results revealed that MSCs are ACE2 or TMPRSS2 negative, indicating that MSCs are free from COVID-19 infection. Moreover, anti-inflammatory and trophic factors like TGF-β, HGF, LIF, GAL, NOA1, FGF, VEGF, EGF, BDNF, and NGF were highly expressed in MSCs, further demonstrating the immunomodulatory function of MSCs. Moreover, SPA and SPC were highly expressed in MSCs, indicating that MSCs might differentiate to AT2 cells (Fig. 5). KEGG pathway analysis showed that MSCs were closely involved in the antiviral pathways (Supplementary Fig. 7).

## DISCUSSION

**Figure 6.**
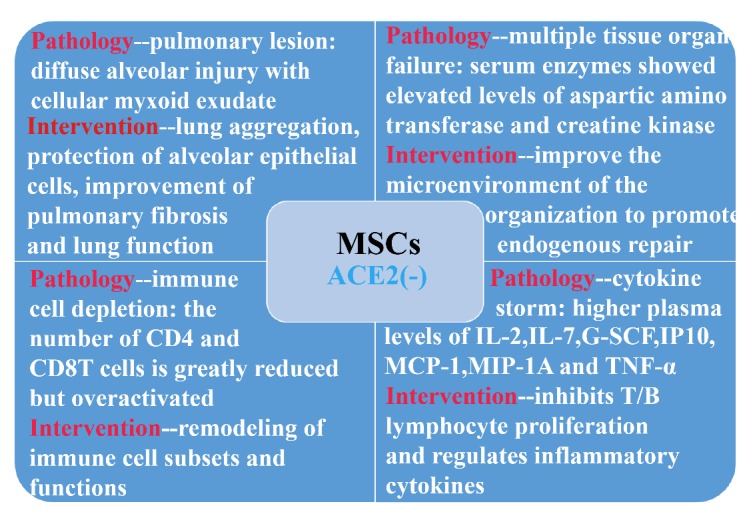
ACE2^-^ MSCs benefit the COVID-19 patients via immunoregulatory function.

Both the novel coronavirus and SARS-2003 could enter the host cell by binding the S protein on the viral surface to the ACE2 on the cell surface [3,5]. In addition to the lung, ACE2 is widely expressed in human tissues, including the heart, liver, kidney, and digestive organs [10]. In fact, almost all endothelial cells and smooth muscle cells in organs express ACE2, therefore once the virus enters the blood circulation, it spreads widely. All tissues and organs expressing ACE2 could be the battlefield of the novel coronavirus and immune cells. This explains why not only all infected ICU patients are suffering from acute respiratory distress syndrome, but also complications such as acute myocardial injury, arrhythmia, acute kidney injury, shock, and death from multiple organ dysfunction syndrome [11] (Fig. 6).

Moreover, the HCoV-19 is more likely to affect older males with comorbidities and can result in severe and even fatal respiratory diseases such as acute respiratory distress syndrome [21], like the critically severe case here. However, the cure of COVID-2019 is essentially dependent on the patient's own immune system. When the overactivated immune system kills the virus, it produces a large number of inflammatory factors, leading to the severe cytokine storms [20]. It suggests that the main reason of these organs damage may be due to virus-induced cytokine storm. Older subjects may be much easier to be affected due to immunosenescence.

Our 10x scRNA-seq survey shows that MSCs are ACE2^-^ and TMPRSS2^-^ (to the best of our knowledge, it is the first time to be reported) and secrete anti-inflammatory factors to prevent the cytokine storm. They have the natural immunity to the HCoV-19. According to the mass cytometry streaming results, the virus infection caused a total function failure of the lymphocytes, even of the whole immune system. MSCs played the vital immune modulation roles to reverse the lymphocyte subsets mainly through dendritic cells. Our previous study showed that co-culture with MSCs could decrease the differentiation of cDC from human CD34+ cells, while increasing the differentiation of pDC through PGE2[22]. Furthermore, the induction of IL-10-dependent regulatory dendritic cells and IRF8-controlled regulatory dendritic cells from HSC were also reported in rats [23,24]. MSCs could also induce mature dendritic cells into a novel Jagged-2-dependent regulatory dendritic cell population [25]. All these interactions with different dendritic cells led to a shift of the immune system from Th1 toward Th2 responses.

Several reports also focused on lymphopenia and high levels of C-reactive protein in COVID-19 patients [20,21]. C-reactive protein is a biomarker with high-sensitivity for inflammation and host response to the production of cytokines, particularly TNFα, IL-6, MCP1 and IL-8 secreted by T cells [26]. However, most mechanistic studies suggest that C-reactive protein itself is unlikely to be a target for intervention. C-reactive protein is also a biomarker of myocardial damage [27].

MSC therapy can inhibit the overactivation of the immune system and promote endogenous repair by improving the microenvironment. After entering the human body through intravenous infusion, part of the MSCs accumulate in the lung, which could improve the pulmonary microenvironment, protect alveolar epithelial cells, prevent pulmonary fibrosis and improve lung function.

As reported by Cao’s team [11], the levels of serum IL-2, IL-7, G-SCF, IP10, MCP-1, MIP-1A and TNF-α in ICU patients were higher than those of normal patients. The cytokine release syndrome caused by abnormally activated immune cells deteriorated the patient’s states which may cause disabled function of endothelial cells, the capillary leakage, the mucus block in lung and finally the respiratory failure. And they could cause even an inflammatory cytokine storm leading to multiple organ failure. The administration of intravenous injection of MSCs significantly improved the inflammation situation in severe COVID-19 patients. Due to its unique immunosuppression capacity, the serum levels of pro-inflammatory cytokines and chemokines were reduced dramatically which attracted less mononuclear/ macrophages to fragile lung, while inducing more regulatory dendric cells to the inflammatory tissue niche. Moreover, the increased IL-10 and VEGF promoted the lung repair. Ultimately, the patients with severe COVID-19 pneumonia survived the worst condition and entered recovery.

Therefore, the fact that the transplantation of MSCs improved the outcome of COVID-2019 patients may be due to regulating inflammatory response and promoting tissue repair and regeneration.

## Supplementary Materials

The Supplemenantry data can be found online at: www.aginganddisease.org/EN/10.14336/AD.2020.0228.
