# Supplementary material for: Transplantation of ACE2- Mesenchymal Stem Cells Improves the Outcome of Patients with COVID-19 Pneumonia
Source: Aging Dis. 2020 Mar 9;11(2):216–28. doi: 10.14336/AD.2020.0228 (PMC7069465; doi:10.14336/AD.2020.0228)
Supplement: Supplementary file 1 — The Supplemenantry data can be found online at: www.aginganddisease.org/EN/10.14336/AD.2020.0228. [file AD-11-2-216-s.pdf]

SUPPLEMENTARY DATA

Transplantation of ACE2$^-$ Mesenchymal Stem Cells Improves the Outcome of Patients with COVID-19 Pneumonia

Zikuan Leng[1,5,#], Rongjia Zhu[2,#], Wei Hou[3,#], Yingmei Feng[3,#], Yanlei Yang[4], Qin Han[2], Guangliang Shan[2], Fanyan Meng[1], Dongshu Du[1], Shihua Wang[2], Junfen Fan[2], Wenjing Wang[3], Luchan Deng[2], Hongbo Shi[3], Hongjun Li[3], Zhongjie Hu[3], Fengchun Zhang[4], Jinming Gao[4], Hongjian Liu[5*], Xiaoxia Li[6], Yangyang Zhao[2], Kan Yin[6], Xijing He[7], Zhengchao Gao[7], Yibin Wang[7], Bo Yang[8], Ronghua Jin[3*], Ilia Stambler[9,10,11], Lee Wei Lim[9,10,12], Huanxing Su[9,10,13], Alexey Moskalev[9,10,14], Antonio Cano[9,10,15], Sasanka Chakrabarti[16], Kyung-Jin Min[9,10,17], Georgina Ellison-Hughes[9,10,18], Calogero Caruso[9,10,19], Kunlin Jin[9,10,20*], Robert Chunhua Zhao[1,2,9,10*]

© 2020. Leng Z et al. Published online at http://www.aginganddisease.org/EN/10.14336/AD.2020.0228.

SUPPLEMENTARY DATA

SUPPLEMENTARY METHODS

Supplementary 1: The method of Mass Cytometry of peripheral blood mononuclear cells (PBMC)

Sample preparation for mass cytometry

PBMC samples were collected from COVID-19 infected patients treated with MSCs transplantation at baseline and on Day 6, and PBMC from a healthy donor were set as the control group. All samples were cultured with 2 μM cisplatin (195-Pt, Fluidigm) for 2 minutes before quenching with CSB (Fluidigm) to identify the viability using mass cytometry analysis. A Fix-I buffer (Fluidigm) was then used to fix cells for 15 min at room temperature, followed by washing three times with phosphate buffer solution (PBS).

Mass cytometry antibody staining and CD45 barcoding

Three samples from the healthy donor, the patient at baseline and Day 6 were stained with CD45 antibodies that were labeled with different metal tags (89, 141 and 172) to minimize internal cross reaction between samples. MaxPar × 8 Polymer Kits (Fluidigm) were used to conjugate with purified antibodies (listed in Supplemental Table 1). All metal-conjugated antibodies were titrated for optimal concentrations before use. Cells were counted and diluted into 1×10^6 cells per milliliter in PBS and underwent permeabilization with 80% methanol for 15 minutes at 0°C. After triple washes in CSB, cells were cultured with antibodies in a total 50 μL CSD for 30 min in RT, triple washed in CSB and incubated with 0.125 μm intercalator in fix and perm buffer (Fluidigm) at 4 °C overnight.

Data acquisition in Helios

After cultured with intercalator, cells were washed three times with ice cold PBS and three times with deionized water. Prior to acquisition, samples were resuspended in deionized water containing 10% EQ 4 Element Beads (Fluidigm) and cell concentrations were adjusted to 1×10^6 cell/ml. Data acquisition was performed on a Helios mass cytometer (Fluidigm). The original FCS data were normalized and .fcs files for everyone were collected.

CyTOF Data Analysis

All .fcs files were uploaded into Cytobank, data cleaning and populations of single living cells were exported as .fcs files for further analysis. Files were loaded into R (http://www.rstudio.com), arcsinh transform was performed to signal intensities of all channels. PhenoGraph analysis was performed.

Supplementary 2: The method of the 10 x RNA-seq survey

Materials and reagents

All supplies and reagents were of the highest grade commercially available. The 0.20 μm-filters, dishes and tubes were purchased from Corning (NY, USA). CD105, CD90, CD44 and CD45 antibodies for the flow cytometry were purchased from Miltenyi Biotec (Bergisch gladbach, Germany). DMEM/F12, fetal bovine serum (FBS), GlutaMAX™-I, TrypLE™ Express, and penicillin and streptomycin antibiotics were purchased from Gibco (California, USA). All other reagents were analytical grade and required no further purification.

Supplementary Table 1. Antibodies used in the Mass cytometry analysis.

Antigen	Symbol and Mass	Antibody Clone	Source
CD45	89Y	HI30	Fluidigm
CD45	141Pr	HI30	Fluidigm
CD19	142Nd	HIB19	Fluidigm
CD5	143Nd	UCHT2	Fluidigm

© 2020. Leng Z et al. Published online at http://www.aginganddisease.org/EN/10.14336/AD.2020.0228.

SUPPLEMENTARY DATA

CCR5	144Nd	NP-6G4	Fluidigm
CD4	145Nd	RPA-T4	Fluidigm
CD45RA	146N	HI100	Biolegend
CD20	147Sm	H1	Fluidigm
CD14	148Nd	RMO52	Fluidigm
CD56	149Sm	NCAM16.2	Fluidigm
CD11c	150Nd	Bu15	Biolegend
CD16	151Eu	3G8	Biolegend
TNFα	152Sm	MAb11	Fluidigm
CD62L	153Eu	DREG-56	Fluidigm
IL-1β	154Sm	Polyclonal	Abcam
CD27	155Gd	L128	Fluidigm
CXCR3	156Gd	G025H7	Fluidigm
IFN-r	158Gd	B27	Fluidigm
CCR7	159Tb	G043H7	Fluidigm
CD28	160Gd	CD28.2	Fluidigm
CD25	161Dy	BC96	biolegend
CD8	162Dy	RPA-T8	Fluidigm
TGFβ	16Dy	TW46H10	Fluidigm
CD45RO	164Dy	UCHL1	Fluidigm
IL-12	165Ho	Polyclonal	Abcam
IL-10	166Er	JES3-9D7	Fluidigm
IL-6	167Er	MQ2-13A5	Biolegend
CD206	168Er	15-2	Fluidigm
CD24	169Tm	ML5	Fluidigm
CD3	170Er	UCHT1	Fluidigm
CD68	171Yb	Y1/82A	Fluidigm
CD45	172Yb	HI30	biolegend
HLA-DR	173Yb	L243	Fluidigm
IL-4	174Yb	MP4-25D2	Biolegend
CD127	176Yb	A019D5	Fluidigm
CD11b	209Bi	ICRF44	Fluidigm

Cell culturing

The mesenchymal stem cells were cultured in DMEM/F12 medium supplemented with 2% FBS, 2% GlutaMAX™-I, 1% antibiotics and 2 mM GlutaMAX™-I at 37°C with 5% CO_2. After three passages, MSCs were immune-phenotyped by flow cytometry for the following surface markers: CD105, CD90, CD73, CD29, HLA-DR, CD44, CD14 and CD45 (all antibodies from BD Pharmingen, San Jose, USA). And MSCs were tested for adipogenic, chondrogenic and osteogenic differentiation to identify their characters.

Cell preparation and Library construction

© 2020. Leng Z et al. Published online at http://www.aginganddisease.org/EN/10.14336/AD.2020.0228.

SUPPLEMENTARY DATA

Cell count and viability were examined by microscope after 0.4% trypan blue coloring. When the viability was no lower than 80%, the library construction was performed. Library was constructed using the Chromium controller (10 x Genomics, Pleasanton, CA). Briefly, single cells, reagents and Gel Beads containing barcoded oligonucleotides were encapsulated into nanoliter-sized GEMs (Gel Bead in Emulsion) using the GemCode technology. Lysis and barcoded reverse transcription of polyadenylated mRNA from single cells were performed inside every GEM. Post RT-GEMs were cleaned up and cDNA were amplified. cDNA was fragmented and fragment ends were repaired, as well as A-tailing was added to the 3' end. The adaptors were ligated to fragments which were double sided SPRI selected. Another double sided SPRI selecting was carried out after sample index PCR. Quality control-pass libraries were sequenced. The final library was quantitated in two ways: determining the average molecule length using the Agilent 2100 bioanalyzer instrument; and quantifying the library by real-time quantitative PCR.

Analysis of single-cell transcriptomics data

The reads were demultiplexed by using the Cell Ranger Single Cell Software Suite (v3.1.0, 10 x Genomics) and R package Seurat (v3.1.0). The number of genes, unique molecule identifier (UMI) counts and percentage of mitochondrial genes were examined to identify outliers. Principal component analysis was used for dimensionality reduction. U-MAP was then used for two-dimensional visualization of the results. DEGs were identified with the FindConservedMarkers function in Seurat by parameters of logfc.threshold >0.25, minPct>0.25 and Padj≤0.05. KEGG pathways with FDR ≤0.05 were considered to be significantly enriched.

Supplementary 3: The detailed diagnosis and treatment procedures for the critically severe patient

On the evening of January 22, 2020, a 65-year-old man presented to the emergency department of Beijing YouAn Hospital, Beijing, with a 2-day history of cough, sputum and subjective fever. The patient wore a mask in the hospital. He disclosed to the physician that he had traveled in Wuhan, China, from December 31, 2019 to January 20, 2020 and returned to Beijing on January 20. Apart from a 10-year history of hypertension with the highest blood pressure of 180/90 mmHg ever, the patient had no other specific medical history. The physical examination showed a body temperature of 37.8, blood pressure of 138/85 mmHg, pulse of 85 beats per minute, respiratory rate of 19 breaths per minute. Lung auscultation revealed rhonchi. A blood routine examination was arranged urgently, and the result revealed that the white-cell count and absolute lymphocyte count were 4.9×10^9/L (reference range $(3.5\sim9.5) \times 10^9$/L) and 0.94×10^9/L (reference range $(1.1\sim3.2) \times 10^9$/L), respectively (Table 1). According to the COVID-19 guidance released by the National Health Commission of China, the physician gave him a diagnosis of a suspected COVID-19 case and asked him to undergo medical isolation observation in the hospital. Meantime, the doctor collected his oropharyngeal swab specimen.

On January 23, 2020, the RT-PCR assay confirmed that the patient's specimen tested positive for HCoV-19. Then the patient was admitted to an airborne-isolation unit in Beijing YouAn Hospital for clinical observation. He had no dyspnea. His consciousness was clear, and the diet and sleep were normal since he became sick. A chest computed tomography (CT) was reported as showing no evidence of infiltrates or abnormalities. The admitting diagnoses were new coronary pneumonia (common type) and hypertension III. The patient received no special care except the irbesartan, which was taken all through the treatment period.

On January 24 to January 29, the patient's vital physical signs remained largely stable, apart from the development of intermittent fevers and shortness of breath. During this time, the patient received antipyretic therapy including 15 ml of ibuprofen suspension every 6 hours and 650 mg of acetaminophen every 6 hours. From January 26, the patient also received antiviral therapy including lopinavir and ritonavir twice a day, with the amount of 400 mg and 100 mg each time, respectively.

On January 30, the patient felt severe shortness of breath and appeared fatigued. The oxygen saturation values measured by pulse oximetry decreased to as low as 91% while he was breathing ambient air. Auscultation rhonchi

© 2020. Leng Z et al. Published online at http://www.aginganddisease.org/EN/10.14336/AD.2020.0228.

became worse in the middle of the double sides of the lung. An urgent chest CT clearly showed evidence of pneumonia, ground-glass opacity, in the middle lobes of the right and left lung. The other positive results of laboratory tests included the C-reactive protein rise to 105.5 g/L (reference range < 3 g/L), but the absolute lymphocyte count decreased to 0.60 × 10^9/L. The potassium concentration went down to 2.74 mmol/L (reference range 3.5-5.5 mmol/L). The doctors decided to change the diagnosis to COVID-19 (critically severe type), and the patient was admitted to ICU unit. More treatments were conducted consisting of mask oxygen supplementation (5 liters per minute), electrocardiograph monitoring, potassium chloride sustained release tablets (oral, 500 mg per time, 3 times per day) and more glucose and amino acid injection. Finally, the discomfort was released, and the oxygen saturation increased to 95%.

On January 31, the shortness of breath even got worse under the oxygen supplementation. The doctor speeded up the oxygen airflow to 10 liters per minute. After the patient signed an agreement to perform the MSCs transplantation, 100 ml of normal saline including 6 × 10^7 MSCs was intravenously injected into the patient, and no adverse events were observed in association with the infusion.

On February 1 and 2, the patient did not feel better. The third chest CT revealed that the pneumonia got worse. On February 1, the levels of C-reactive protein were 191.0 g/L, and the absolute lymphocyte count decreased badly to 0.23 × 10^9/L. The laboratory results showed that his liver and myocardium were very likely to be affected. The electrocardiograph monitoring showed the blood pressure, heart rate, respiratory rate and oxygen saturation were 138/80 mmHg, 95 bpm, 33 bpm and 93% under the mask oxygen supplementation of 10 liters per minute. The doctors informed the patient's families of a critical condition.

However, the patient felt better on February 3, for instance, the shortness of breath was significantly recovering. On February 4, the C-reactive protein decreased to 13.6 g/L, and the absolute lymphocyte count rose to 0.58 × 10^9/L, which indicated that the patient was recovering rapidly. The indexes of liver and myocardium function recovered. Both fever and shortness of breath disappeared on February 5. He was rolled out of ICU. On February 9, the fourth chest CT confirmed that the pneumonia was disappearing. On February 13, the C-reactive protein concentration was 10.1 g/L, and the absolute lymphocyte count was 0.93 × 10^9/L. Up to now, the patient felt much better.

Supplementary 4. More results of the 10 x RNA-seq survey

Flow cytometry analysis

The PI staining results showed that 91.60% of the total cell population was alive, and the cells were: $CD105^+$, $CD90^+$, $CD73^+$, $CD44^+$, $CD29^+$, $CD14^-$ and $CD45^-$ (Supplementary Fig. 1).

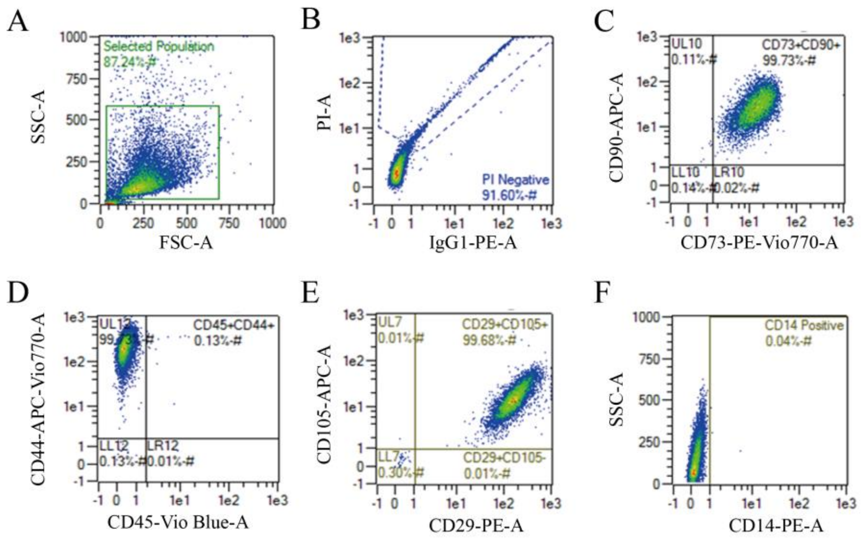

Supplementary Figure 1. Flow cytometry evaluation of transplanted MSCs (A) Single cells (87%) were gated firstly. (B) Live cells (91% of the single cells) were enrolled. (C-F) 99% of selected cells were $CD105^+$, $CD90^+$, $CD73^+$, $CD44^+$, $CD29^+$, $CD14^-$ and $CD45^-$.

© 2020. Leng Z et al. Published online at http://www.aginganddisease.org/EN/10.14336/AD.2020.0228.

SUPPLEMENTARY DATA

The overview of the survey

A deep transcriptional state map of MSCs and gene expression at single-cell level was generated after the performance of 10× Genomics high throughput of RNA sequencing. The 12,500 cells were acquired in the survey, leading to 881,215,280 raw reads totally. The median number of genes and UMIs detected per cell were 4,099 and 23,971, respectively (Supplemental Figure 2). The sequencing saturation rate was 72.9%, which met the scRNA-seq requirements.

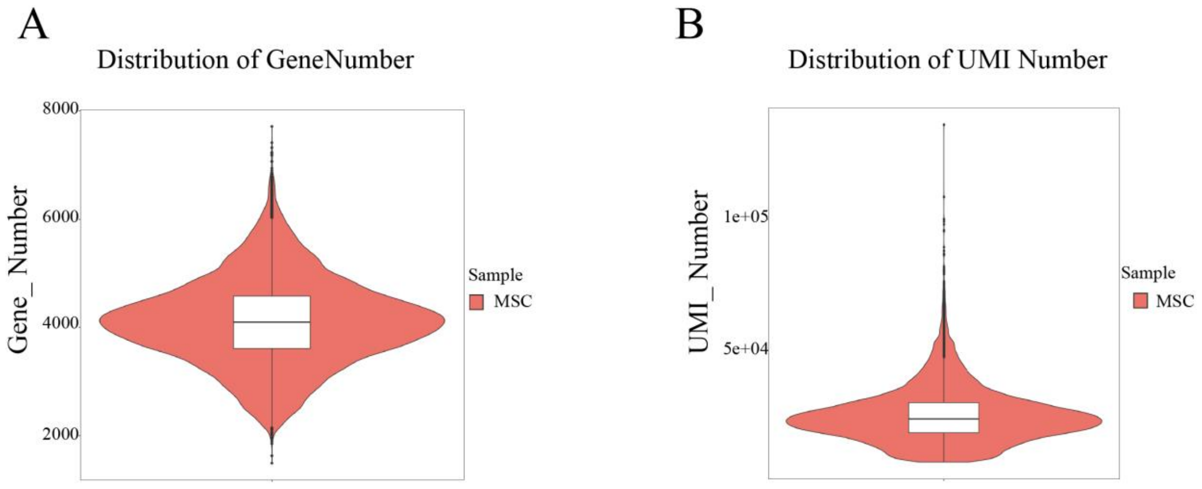

Supplementary Figure 2. In the 10 x RNA-seq survey, the median number of genes and UMIs detected per cell were 4,099 (A) and 23,971 (B) as showed in the violin distribution.

MSCs marker genes expression

The scRNA-seq showed that the MSCs highly expressed ENG (CD105), THY1 (CD90), and NT5E (CD73). However, the expression of PTPRC (CD45), CD34, CD14, CD19, and HLA-DR was nearly undetected in the cells (CD45 was the only one shown in Supplemental Figure 3). The results were in accordance with the flow cytometry analysis. In Supplemental Figure 3, one point meant one cell, and red and gray color represented high expression and low expression, respectively.

© 2020. Leng Z et al. Published online at http://www.aginganddisease.org/EN/10.14336/AD.2020.0228.

SUPPLEMENTARY DATA

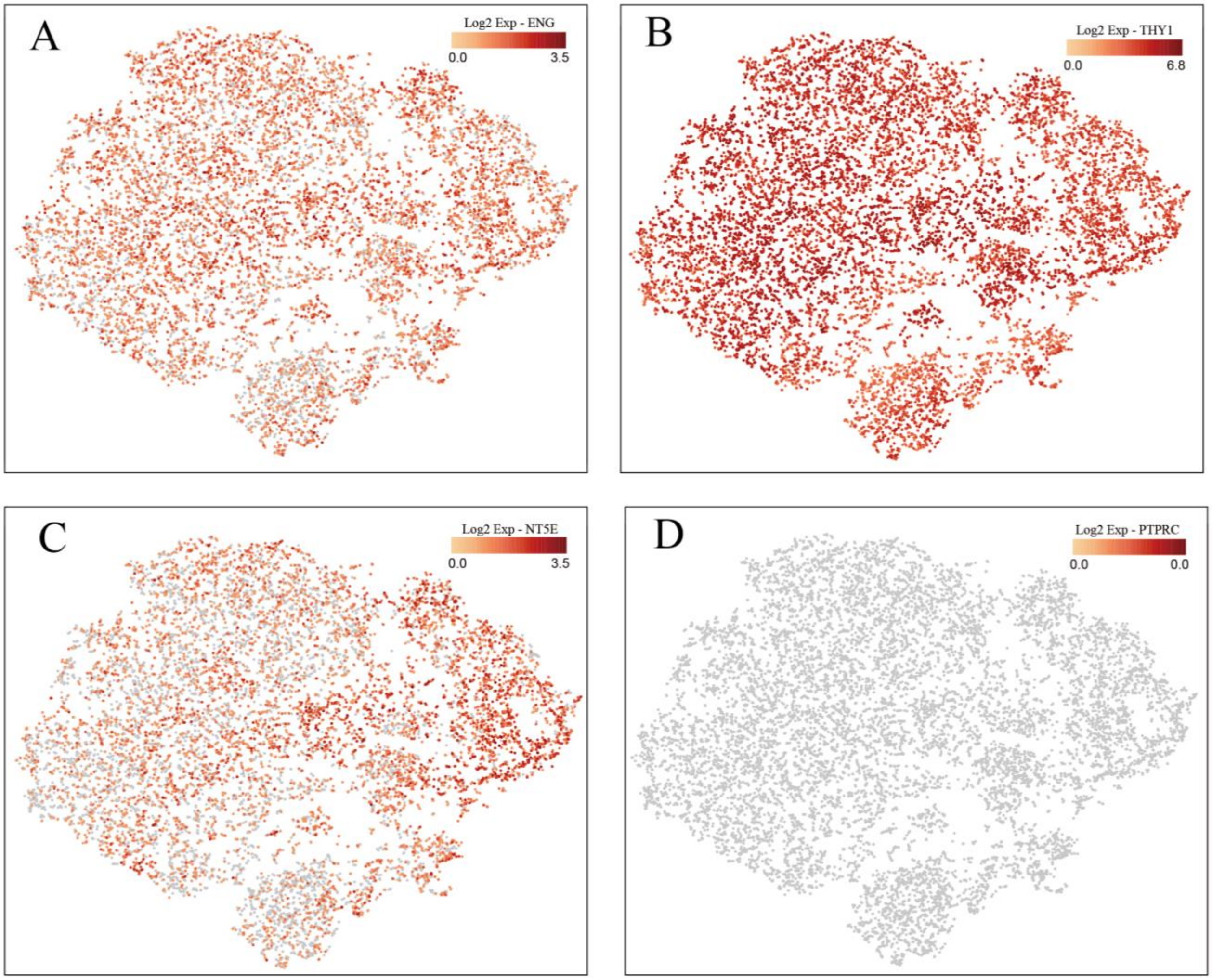

Supplementary Figure 3. MSCs marker genes expression by 10 x scRNA-seq analysis. (A) $CD105^+$, (B) $CD90^+$, (C) $CD73^+$, and (D) $CD45^-$.

ACE2 gene expression and DEGs between $ACE2^+$ MSC and $ACE2^-$ MSC

Only one of the 12,500 cells was $ACE2^+$ as shown in Supplemental Figure 4A. Furthermore, the top 60 DEGs between the $ACE2^+$ MSC and the other nearby $ACE2^-$ MSC were shown in Supplemental Figure 4B. It is revealed that the $ACE2^+$ MSC tended to generate pro-inflammatory function by secreting IL-8, IL-6 and so on, while $ACE2^-$ MSC tended to generate anti-inflammatory effect by secreting BDNF and other factors.

© 2020. Leng Z et al. Published online at http://www.aginganddisease.org/EN/10.14336/AD.2020.0228.

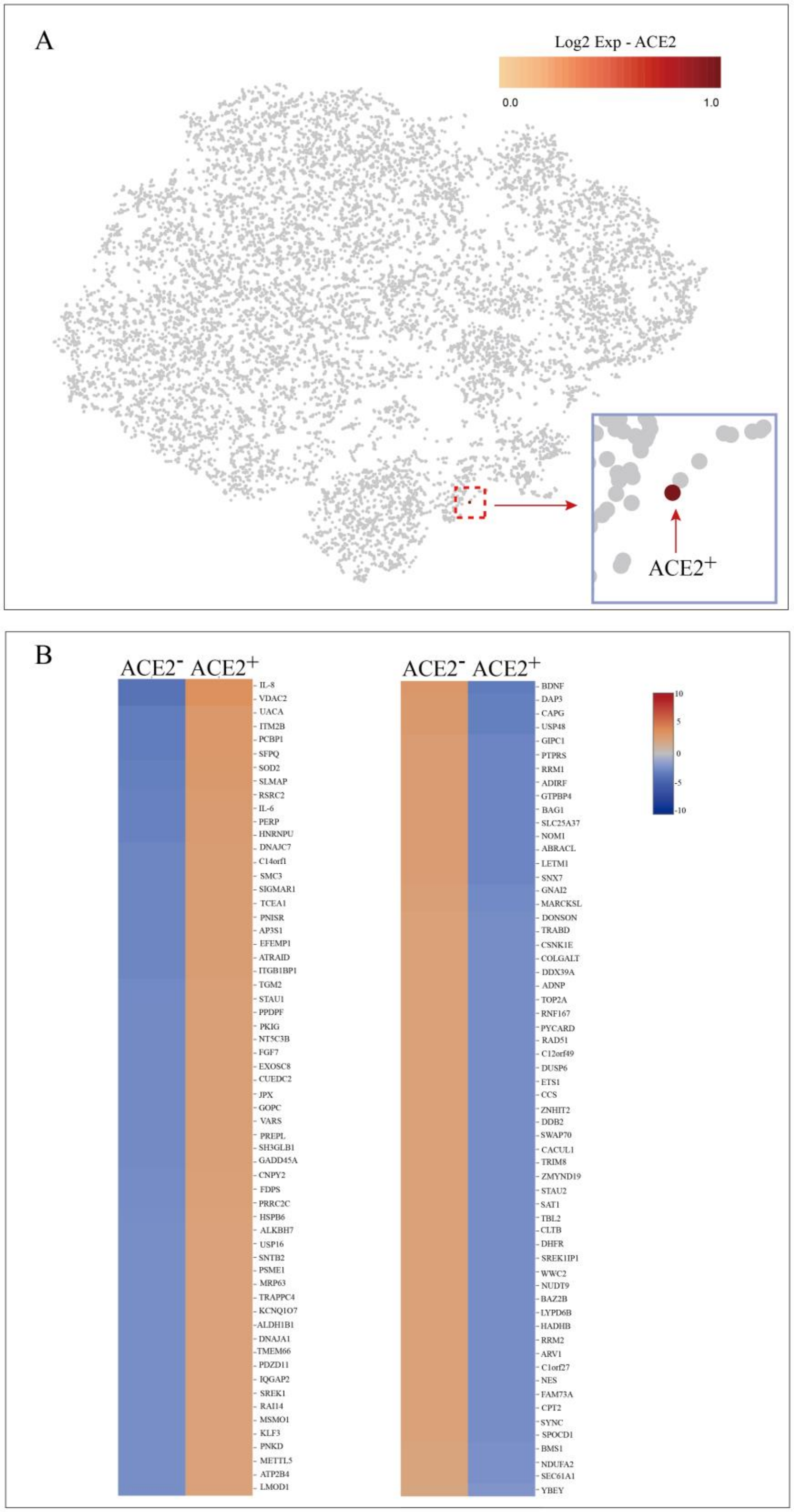

Supplementary Figure 4. (A) ACE2 gene expression in MSCs. (B) top 60 DEGs between one ACE2$^+$ MSC and one ACE2$^-$ MSC.

© 2020. Leng Z et al. Published online at http://www.aginganddisease.org/EN/10.14336/AD.2020.0228.

SUPPLEMENTARY DATA

TMPRSS2 gene expression and DEGs between TMPRSS2 + MSC and TMPRSS2 − MSC

Only seven of the 12,500 cells were TMPRSS2$^+$ as shown in Supplemental Figure 5A. Furthermore, the top 60 DEGs between the TMPRSS2$^+$ MSC and the other seven nearby TMPRSS2$^-$ MSC were shown in Supplemental Figure 5B.

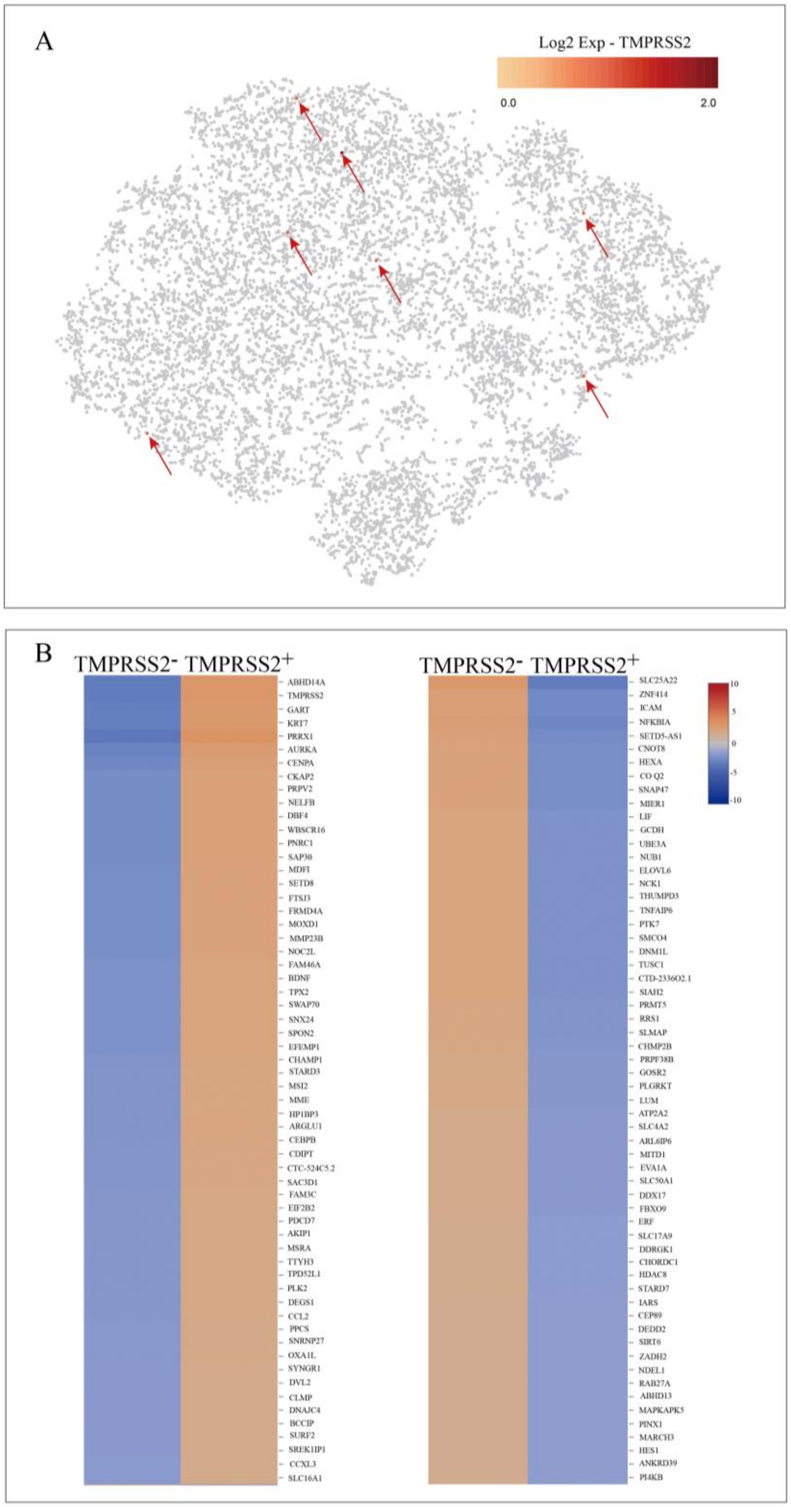

Supplementary Figure 5. (A) TMPRSS2 gene expression in MSCs. (B) top 60 DEGs between seven TMPRSS2$^+$ MSCs and seven TMPRSS2$^-$ MSCs.

© 2020. Leng Z et al. Published online at http://www.aginganddisease.org/EN/10.14336/AD.2020.0228.

SUPPLEMENTARY DATA

Kyoto Encyclopedia of Genes and Genomes (KEGG) analysis

KEGG pathway analysis demonstrated diseases mainly related to viral infectious diseases, cancers and endocrine and metabolic disorders (1727 genes, 1605 genes and 1384 genes, respectively). Organismal systems mainly related to endocrine and immune systems (1578 genes and 748 genes, respectively) (Supplemental Figure 6). Four enriched KEGG pathways were also involved in viral infection (Supplemental Figure 7).

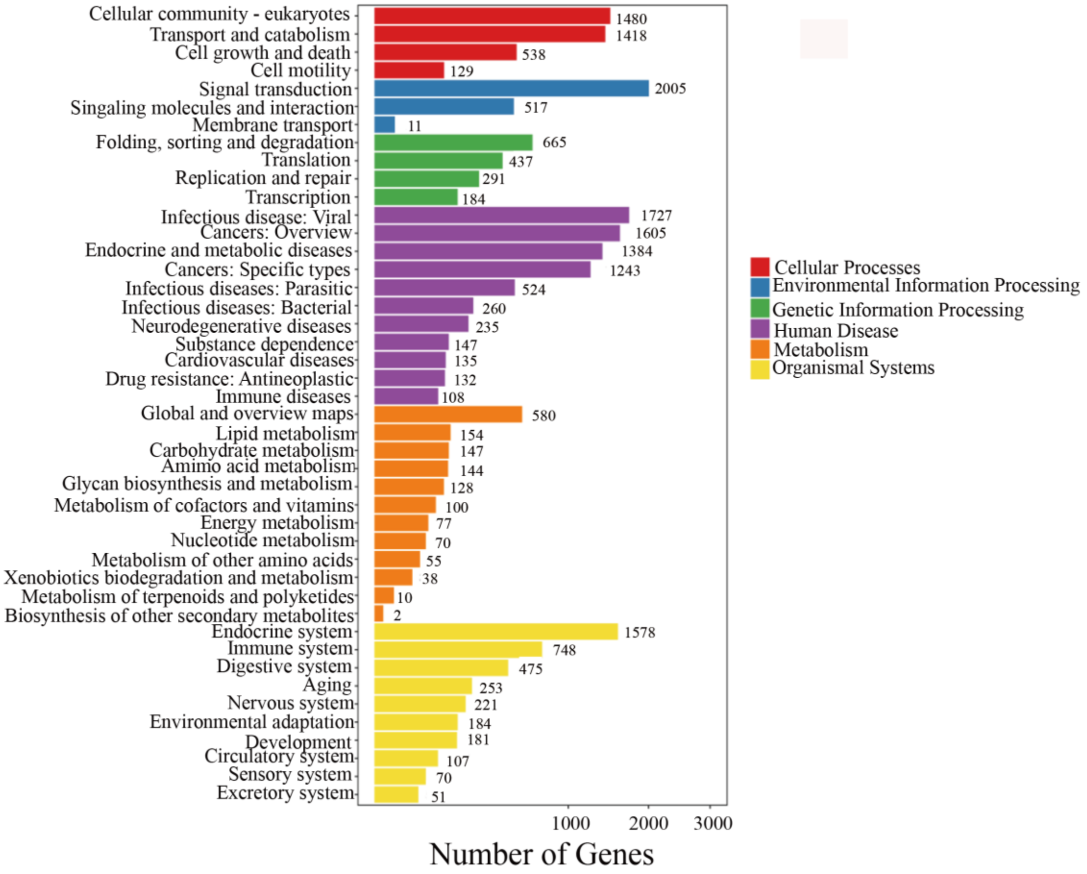

Supplementary Figure 6. Kyoto Encyclopedia of Genes and Genomes (KEGG) analysis revealed that many gene expressions of MSCs were related with endocrine and immune systems.

© 2020. Leng Z et al. Published online at http://www.aginganddisease.org/EN/10.14336/AD.2020.0228.

SUPPLEMENTARY DATA

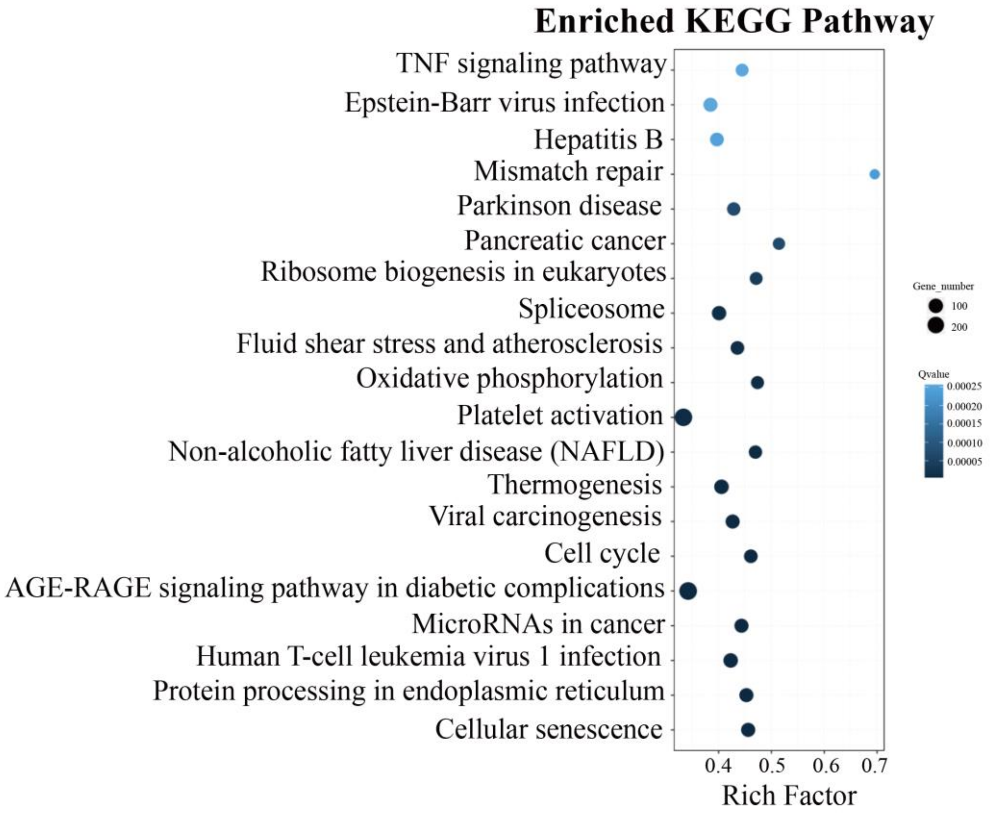

Supplemental Figure 7. Four enriched KEGG pathways were also involved in viral infection, namely Epstein-Barr virus infection, Hepatitis B, viral carcinogenesis and human T-cell leukemia virus 1 infection.

Supplementary 5. Serum Cytokine and chemokine measurement

To detect the effect of MSCs on the production of cytokines or chemokines in the serum of enrolled patients, cytokines TNF-α, IL-10, VEGF-A and chemokines IP-10 were measured using Human Cytokine/Chemokine/Growth Factor Panel A Magnetic Bead 96-Well Plate Assay (EMD Millipore, Billerica, MA USA) according to the manufacturer's instructions. Run plate on Luminex® 200™ with xPONENT® software. These samples were from patient before and after MSC treatment as well as the control group patients.

© 2020. Leng Z et al. Published online at http://www.aginganddisease.org/EN/10.14336/AD.2020.0228.
